# The Impact of Environmental Gaseous Pollutants on the Cultivable Bacterial and Fungal Communities of the Aerobiome

**DOI:** 10.3390/microorganisms12061103

**Published:** 2024-05-29

**Authors:** Madelaine Mejías, Romina Madrid, Karina Díaz, Ignacio Gutiérrez-Cortés, Rodrigo Pulgar, Dinka Mandakovic

**Affiliations:** 1 GEMA Center for Genomics, Ecology and Environment, Facultad de Ciencias, Ingeniería y Tecnología, Universidad Mayor, Santiago 8580745, Chile; madelaine.mejias@mayor.cl (M.M.); romina.madrid@mayor.cl (R.M.); karina.diaz.c@ug.uchile.cl (K.D.); igutierrezc@estudiante.uc.cl (I.G.-C.); 2 Programa de Doctorado en Ecología Integrativa, Universidad Mayor, Santiago 8580745, Chile; 3 Laboratorio de Genómica y Genética de Interacciones Biológicas (LG2IB), Instituto de Nutrición y Tecnología de los Alimento, Universidad de Chile, Santiago 7830490, Chile

**Keywords:** aerobiome, cultivable community, bacteria, fungi, air pollution, gaseous pollutants, high-throughput sequencing

## Abstract

Understanding air microbial content, especially in highly polluted urban areas, is crucial for assessing its effect on human health and ecosystems. In this context, the impact of gaseous pollutants on the aerobiome remains inconclusive due to a lack of studies separating this factor from other contaminants or environmental factors. In this study, we aimed to experimentally assess the influence of contrasting concentrations of atmospheric gaseous pollutants as isolated variables on the composition of the aerobiome. Our study sites were contrasting Air Quality Index (AQI) sites of the Metropolitan Region of Chile, where nitric oxide (NO) was significantly lower at the low-AQI site than at the high-AQI site, while ozone (O_3_) was significantly higher. Cultivable aerobiome communities from the low-AQI site were exposed to their own pollutants or those from the high-AQI site and characterized using high-throughput sequencing (HTS), which allowed comparisons between the entire cultivable communities. The results showed increased alpha diversity in bacterial and fungal communities exposed to the high-AQI site compared to the low-AQI site. Beta diversity and compositional hierarchical clustering analyses revealed a clear separation based on NO and O_3_ concentrations. At the phylum level, four bacterial and three fungal phyla were identified, revealing an over-representation of Actinobacteriota and Basidiomycota in the samples transferred to the high-AQI site, while Proteobacteria were more abundant in the community maintained at the low-AQI site. At the functional level, bacterial imputed functions were over-represented only in samples maintained at the low-AQI site, while fungal functions were affected in both conditions. Overall, our results highlight the impact of NO and/or O_3_ on both taxonomic and functional compositions of the cultivable aerobiome. This study provides, for the first time, insights into the influence of contrasting pollutant gases on entire bacterial and fungal cultivable communities through a controlled environmental intervention.

## 1. Introduction

Air pollution and the aerobiome (airborne bacteria, fungi, and viruses) have traditionally received public attention for their association with health threats for animals, plants, and especially humans [1,2] due to the constancy and ubiquity of our exposure [3]. Airborne microbes are associated with the particulate matter (PM) of the air that can be deposited into and directly affect different regions of the respiratory tract and may indirectly impact other systems [4,5,6,7]. In addition, increased air pollution in cities directly influences the taxonomic composition and diversity of the aerobiome, as air pollutants create selective pressures for some microorganisms, allowing them to thrive in heightened-pollution environments [8,9,10]. However, most aerobiome studies have primarily examined the correlation between PM and airborne bacterial communities, disregarding the impact of other contaminants. This bias has limited the understanding of the effect of gaseous pollutants on the aerobiome composition, especially on other important members of the aerobiome, such as fungi; thus, studying the relationship between air pollution and the airborne microbial community, in a more ample context, is essential to understand the ecology of aerobiome ecosystems and its influence on the health of living organisms [11].

Air pollutants, collectively assessed in the Air Quality Index (AQI), are composed of a heterogeneous mixture of solid particles and gaseous compounds that primarily include the PM (classified as PM2.5 and PM10, depending on their nominal mean aerodynamic diameters of ≤2.5 and ≤10 μm, respectively), together with ozone (O_3_), sulfur dioxide (SO_2_), nitrogen oxides (NO_x_), and carbon monoxide (CO), among others [12]. As for gaseous pollutants, environmental studies have included them in general atmospheric parameters. For example, Li et al. (2019) [9] analyzed the bacterial community over four seasons in an urban city in China using high-throughput sequencing (HTS). They observed significant correlations between several genera and environmental factors, including O_3_, SO_2_, and CO. Similarly, Fan et al. [13] reported a significantly positive correlation between the microbial diversity of HTS-analyzed samples collected at different levels of air pollution in Beijing, China, and environmental factors such as SO_2_. On the other hand, Núñez and García [14] informed a significant negative correlation between the Firmicutes’ abundance and the NO concentration in the urban environment of Madrid (Spain). Despite their interesting findings, culture-independent approaches have not provided knowledge of the exclusive effects of pollutant gases on the microbial airborne community. Moreover, although culture-independent methods are of great utility in characterizing microbial communities, differentiating viable or metabolically active microbes from non-viable community members is complex [15]. To address this limitation, cultivation has been proposed, which, despite containing only a fraction of the viable microorganisms in the environment, allows for studying the adaptation of viable microorganisms to different environmental stimuli [16]. In this context, gaseous pollutants’ impact on the culturable portion of the airborne bacteriome has also been investigated across contamination levels, revealing a negative correlation between CO concentration and the abundance of culturable bacteria measured using colony-forming unit counts [17]. Yet, this study neither separated the impact of gaseous pollutants from other environmental factors nor examined the entire cultivable community. Therefore, cultivable community interventional studies using HTS could offer a deeper understanding of how pollutants affect the diversity and composition of the entire air’s cultivable microbial community.

In brief, while studies have demonstrated the influence of air pollutants on both the total and culturable portions of airborne bacteria, they have often neglected the inclusion of the fungal community in their analyses. Moreover, studies on the cultivable aerobiome community have relied on colony-forming unit counts to assess the impact of contaminants, overlooking advanced techniques like HTS [17,18]. Lastly, the impact of gaseous pollutants remains inconclusive due to a lack of studies isolating this factor from other contaminants such as PM or environmental factors like humidity and temperature. In this study, our aim was to experimentally assess the influence of contrasting concentrations of atmospheric gaseous pollutants as isolated variables on the entire cultivable bacterial and fungal communities via HTS of the aerobiome of the Metropolitan Region (RM) of Chile, known as one of the most polluted cities in South America [19]. To do so, we sampled the aerobiome from a low-AQI site and either cultured it at the same site or transferred it to a high-AQI site, with or without exposure to atmospheric gaseous pollutants for one week. Next, we characterized the cultivable bacterial and fungal aerobiomes using HTS and analyzed changes in their diversity, taxonomy, and functions, which were associated with variations in the concentration of air-polluting gases. To our knowledge, this is the first study to provide information on the influence of environmental pollutant gases on entire air-cultivable communities of bacteria and fungi through a controlled experimental intervention.

## 2. Materials and Methods

### 2.1. Sampling Sites, Experimental Design, and Environmental Parameters

Two districts in the Metropolitan Region of Chile were chosen based on historical differences in winter gas concentrations: Las Condes, with historically low levels of pollution (the low-AQI site) and Pudahuel, with historically high levels of pollution (the high-AQI site), which is explained by the fact that Pudahuel has fewer green areas and less ventilation than Las Condes, as well as more industrial and transport activity (more sources of fossil fuel combustion activities). Air samples were collected simultaneously at the low-AQI site on 12 August 2022 using AM2050A Portable Planktonic Bacteria Air Samplers (Shanghai, China) and Petri dishes (Wuxi, China) for 5 min to obtain representative cultivable microbial airborne communities from this site and from the same sampling time. To capture a greater diversity of airborne microorganisms, we employed three culture media commonly utilized in aerobiome studies (Nutrient Agar (NA), Luria Bertani (LB), and Tryptic Soy Agar (TSA)) that support the growth of a wide range of microorganisms. Triple-sectioned plates were employed, with each section containing a distinct culture medium. Twelve samples were taken, with half under no gas exposure (“g−”) since they were covered with their respective lids, and the other half under gas exposure (“g+”) since they were covered with gas-permeable membranes (AeraSeal™ film, Sigma-Aldrich, Darmstadt, Germany). Three plates from each condition were “maintained” at the low-AQI site, while the remaining three plates from each condition were “transferred” to the high-AQI site. After seven days in their respective locations (experimentation week from 12 to 19 August 2022), the plates were collected and photographed, and the microbial DNA was extracted for analysis.

The pollutant concentrations and meteorological parameters for each district during the experimentation week were obtained hourly from the Sistema de Información Nacional de Calidad del Aire (SINCA; https://sinca.mma.gob.cl accessed on 21 December 2023). 

### 2.2. DNA Extraction 

For DNA extraction, the surface of each culture medium was carefully scraped with a sterile scalpel to release adhered microorganisms. Four milliliters of sterile PBS were added to each plate to mix colonies from the entire plate. The liquid with suspended microorganisms was transferred to 2-mL microcentrifuge tubes. After centrifugation at 13,000 rpm for 5 min, the supernatant was discarded. The PowerSoil DNA isolation kit (Qiagen, Hilden, Germany) was used, following the manufacturer’s protocol with a modification: reducing the number of beads in tubes to 1/3 for the mechanical rupture of microorganisms. Finally, 50 μL of DNA was obtained from each plate. The DNA integrity was evaluated via electrophoresis in Agilent 2200 TapeStation (Agilent, Santa Clara, CA, USA), and the DNA concentration was measured via fluorescence with Qubit equipment (Thermo Fisher Scientific, Waltham, MA, USA). Then, the DNA was stored at 4 °C until use.

### 2.3. Kingdom Relative Abundance via qPCR

Reactions were carried out on a real-time AiaMx Real-Time PCR System (Agilent) using the Brilliant II SYBR^®^ Green QPCR Master Mix (Agilent). For all samples, 100 ng of DNA was used as the template for qPCR, with universal qPCR bacterial primers 338 F (5′-ACTCCTACGGGAGGCAGCG-3′) and 528 R (5′-ATTACCGCGGCTGCTGG-3′) and fungal primers FF390 (5′-CGATAACGAACGAGACCT-3′) and FR1 (5′-AICCATTCAATCGGTAIT-3′). In brief terms, the bacterial PCR conditions were 95 °C for 2 min, followed by 95 °C for 1 min, 58.7 °C for 30 s, and 72 °C for 1 min for a total of 35 cycles and finally 72 °C for 5 min, while the fungal PCR conditions were 95 °C for 2 min, followed by 95 °C for 1 min, 42.6 °C for 45 s, and 72 °C for 1 min for a total of 35 cycles and finally 72 °C for 5 min. To determine the relative abundances of the genes, the method described by Pfaffl [20] and adapted by Talke [21] was used.

### 2.4. DNA High-Throughput Sequencing and Taxonomic Identification

Microbial DNA was amplified using the bacteria-specific primer sets 515 F (5′-GTGCCAGCMGCCGCGGTAA-3′) and 907 R (5′-CCGTCAATTCCTTTGAGTTT-3′), flanking variable regions V4-V5 of the 16S rRNA gene, and the fungi-specific primer sets ITS5-1737 F (5′-GGAAGTAAAAGTCGTAAGAAGG-3′) and ITS2-2043 R (5′-GCTGCGTTCTTCATCGATGC-3′), flanking the ITS1 region. Sequencing was performed at Novogene Corporation Inc. (Sacramento, CA, USA) on an Illumina NovaSeq platform in an overlapping 2 × 250 bp configuration.

Microbiome bioinformatics were performed with QIIME 2 2022.2. Raw sequence data were demultiplexed and quality-filtered with the minimal quality median set to 30 using the q2-demux plugin, followed by denoising with DADA2 [22] (via q2-dada2). The taxonomy was assigned to amplicon sequence variants (ASVs) using the q2-feature-classifier [23] classify-sklearn naïve Bayes taxonomy classifier against the SILVA v138.99 and UNITE v8.99 reference sequences for bacteria and fungi, respectively. By doing this, we obtained 396 bacterial and 324 fungal ASVs comprising 1,628,799 and 1,652,272 reads, respectively. For analyses, we selected reads that mapped with ASVs that were identified in at least two out of three replicates to analyze data using representative ASVs from each sample. Using this criterion, 113 bacterial and 43 fungal ASVs were included in the analyses. Additionally, to isolate the impact of the pollutant gases between sites on the cultivable communities and exclude the influence of other environmental differential factors between sites, the abundances of ASVs in each gas-exposed community (g+) were standardized based on their counterparts’ non-gas-exposed community (g−) for all microbial analyses. For this, samples with zero (0) relative abundance in the g− condition were replaced with a one (1) since any number divided by one is equal to the number itself; therefore, the abundance difference between g+ and g− was maintained [24,25]. Hence, the input data were the nine abundance g+/g− ratios obtained for each ASV (three g+ replicates and three g− replicates) of each condition. The complete set of raw data was deposited into the SRA experiment database PRJNA1064435. 

### 2.5. Microbial Diversity and Imputed Functional Analysis

Bacterial and fungal alpha diversities (observed features and the Shannon index) were estimated using q2-diversity after the samples were rarefied to 112,000 and 106,000 bacterial and fungal sequences per sample, respectively. Beta diversity was evaluated via a principal coordinates analysis (PCA) with Bray–Curtis distance. 

The functional assignment of the taxonomic data obtained was performed in R v4.3.0, using the Microeco (Microbial Community Ecology Data Analysis) package, version 1.3.1 [26]. The functional assignment of bacterial and fungal communities was performed using the databases FAPROTAX v1.2.6 [27] and FungalTraits v0.03 [28], respectively.

### 2.6. Statistical Analyses

Pollutants and meteorological parameters were compared between sites with a Mann–Whitney test. DNA concentrations between samples were compared with a Kruskal-Wallis test. Alpha microbial diversity (observed features and Shannon index) comparisons between conditions were evaluated with a Mann–Whitney test. The beta diversity (Bray–Curtis dissimilarity) between groups was evaluated through a permutational analysis of multivariate dispersions (PERMDISP) and a permutation-based multivariate analysis of variance (PERMANOVA). A microbial taxonomic and imputed functional Z-score hierarchical clustering analysis was based on the Euclidean distance of the ASV or the imputed functional composition. Taxonomic and imputed functional differences between conditions were compared with a Mann–Whitney test. Differences between the groups were considered significant if *p* was <0.05. Statistical analyses were performed with the GraphPad Prism 10 software and the Microbiome Analyst platform [29].

## 3. Results

### 3.1. The Metropolitan Region of Chile Exhibits Contrasting Environmental Landscapes

**Figure 1:**
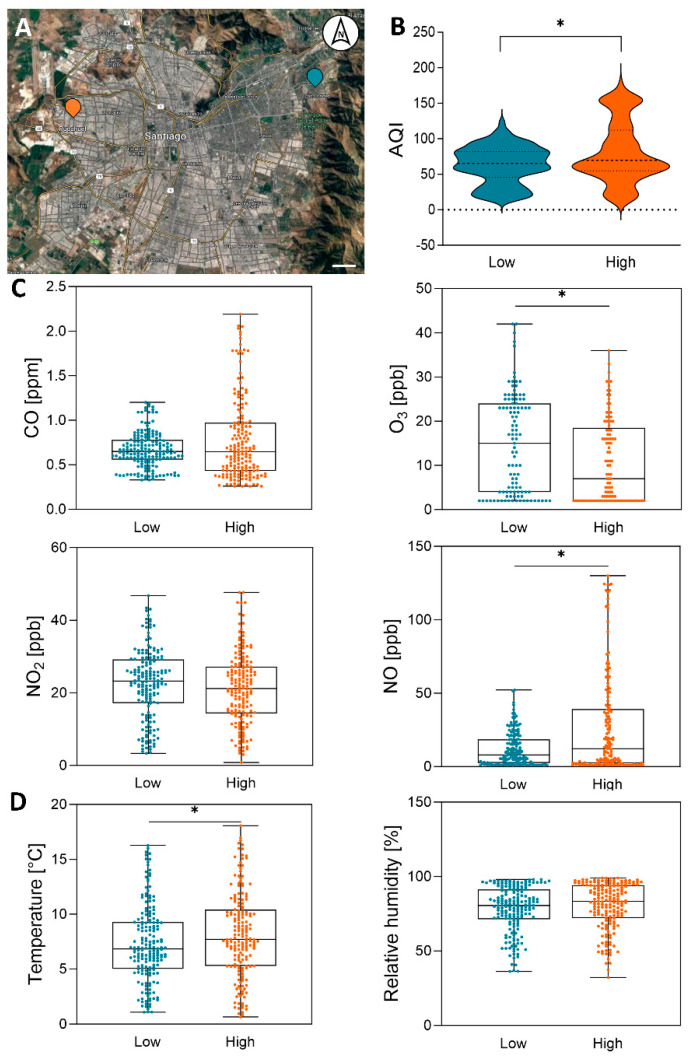
Study sites and environmental parameters. (**A**) Regional context of the Metropolitan Region of Chile. Study sites on a west-to-east transect: “Pudahuel” (orange) and “Las Condes” (blue). The map was obtained from Google Maps. Scale: 2 km. (**B**) Violin plots of the Air Quality Index (AQI) data from the low- and high-AQI sites during the experimentation week (12–19 August 2022) obtained from Sistema de Información Nacional de Calidad del Aire (SINCA; https://sinca.mma.gob.cl/, accessed on 21 December 2023). The dashed bold lines represent the median. The dotted lines at the tops and bottoms represent the 75th and 25th quartiles, respectively. The asterisks indicate statistically significant differences between sites (Mann–Whitney; *p* < 0.05). (**C**) Box plots of the individual data from gas contaminants during the experimentation week obtained from SINCA. The horizontal lines within boxes represent the medians. The tops and bottoms of the boxes represent the 75th and 25th quartiles, respectively. The asterisks indicate statistically significant differences between sites (Mann–Whitney; *p* < 0.05). (**D**) Box plots of the meteorological data during the experimentation week obtained from SINCA. The horizontal lines within boxes represent the medians. The tops and bottoms of the boxes represent 75th and 25th quartiles, respectively. The asterisks indicate statistically significant differences between sites (Mann–Whitney; *p* < 0.05).

The Metropolitan Region of Chile, particularly during winter, is characterized as one of the most air-polluted regions of South America. Despite this, there are notable variations in air pollutant concentrations among districts. For instance, “Pudahuel” and “Las Condes,” positioned in a west-to-east transect of the Metropolitan Region (Figure 1A), exhibit historical differences in Air Quality Index (AQI) values (Figure S1A), being the highest in Pudahuel (the “high” AQI site) and the lowest in Las Condes (the “low” AQI site). For this reason, these contrasting districts were selected to study how their differences relate to their aerobiome composition. We sampled the cultivable microbial community during a representative winter week in the southern hemisphere. As shown in Figure 1B, the results indicated that, as expected, during the study week, the low-AQI site showed lower AQI values than the high-AQI site. In addition, we collected weekly data on individual atmospheric gases such as carbon monoxide (CO), nitric oxide (NO), ozone (O_3_), and nitrogen dioxide (NO_2_) (Figure 1C), together with environmental parameters such as temperature and relative humidity (Figure 1D). The results indicated that NO and temperature were significantly lower at the low-AQI site than at the high-AQI site, while O_3_ was significantly higher. No changes in CO and NO_2_ concentrations or relative humidity were observed between the sites.

### 3.2. The Microbial Diversity of the Aerobiome-Cultivable Communities Is Modulated by Environmental Gaseous Pollutants

**Figure 2:**
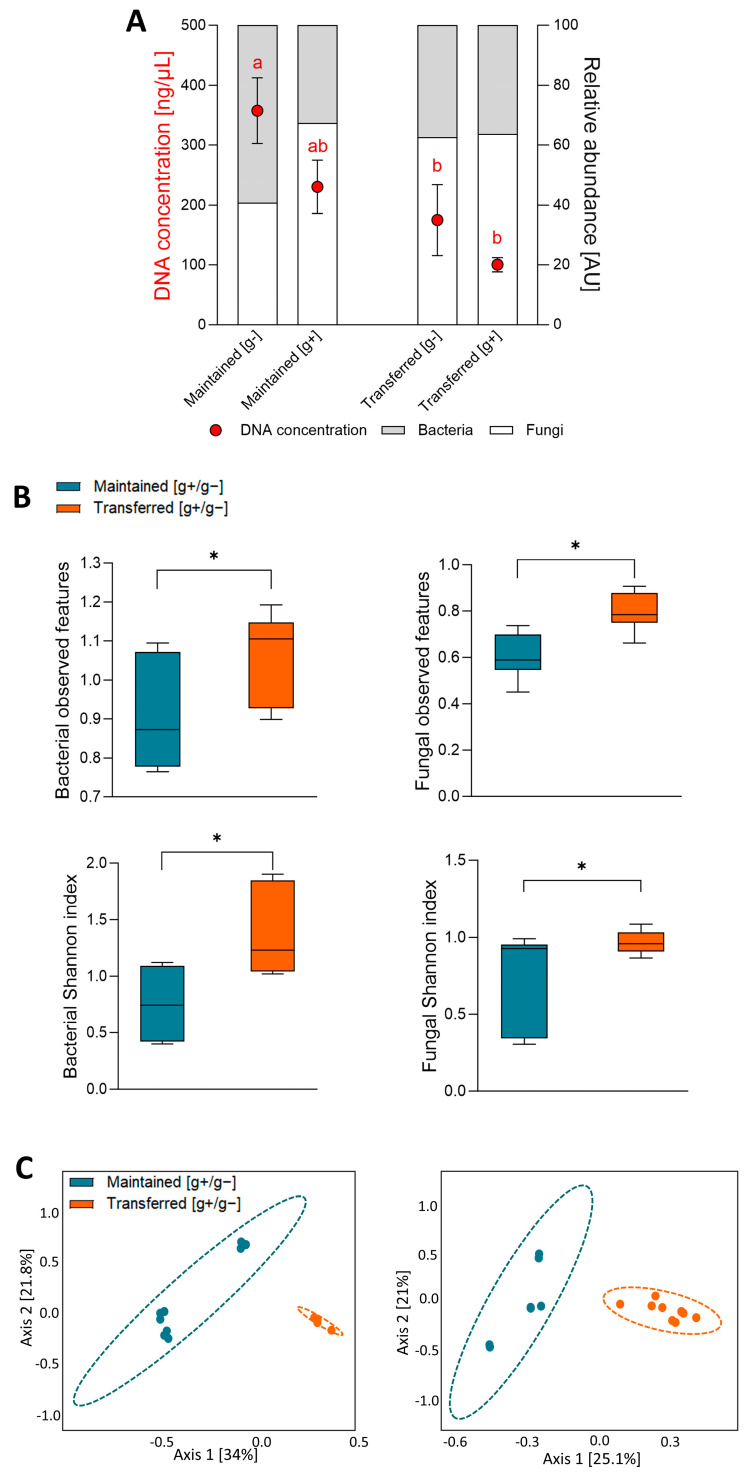
Kingdom-level relative abundance and microbial diversity of the cultivable communities. (**A**) DNA concentration (red dots) and relative abundance of bacteria (gray) and fungi (white) measured via qPCR from the cultivable communities. Different letters indicate statistically significant differences between samples (Kruskal-Wallis; *p* < 0.05). (**B**) Alfa diversity. Bacterial and fungal observed features and Shannon index using the g+/g− ratio data. The box plots’ horizontal lines within boxes represent the medians. The tops and bottoms of the boxes represent the 75th and 25th quartiles, respectively. The asterisks indicate statistically significant differences between samples (Mann–Whitney; *p* < 0.05). (**C**) Beta diversity. PCA of bacterial (left) and fungal (right) samples using the g+/g− ratio data. For bacteria, Bray–Curtis index; PERMANOVA F-value: 7.5; R-squared: 0.32; *p*-value < 0.001. For fungi, Bray–Curtis index; PERMANOVA F-value: 5.13; R-squared: 0.24; *p*-value < 0.001. Maintained [g+/g−] (blue): samples collected from the low-AQI site and grown at this same site; transferred [g+/g−] (orange): samples collected at the low-AQI site and grown at the high-AQI site. g−: no gas exposure; g+: gas exposure.

Considering that the differences in AQI observed between our study sites can be partly explained by differences in some particular gases, cultivable microbial communities collected from the low-AQI site were either “maintained” at the low-AQI site or “transferred” to the high-AQI site and exposed (g+) or not (g−) to the environmental gases of each site. Our results showed that, after the experimentation week, morphological differences were observed between the cultivable microbial communities under both the “maintained” (Figure S2A) and “transferred” conditions (Figure S2B). These visible differences manifested in the abundance, size, and color of bacterial and fungal colonies, attributed to variations in gaseous pollutants and meteorological factors between the sites. To obtain a more specific and quantitative understanding of the visual observations, we conducted a molecular analysis of the microbial communities. Initially, we measured the total DNA concentration in the cultivable communities and found no significant difference between g+ and g− communities maintained at the low-AQI site (Figure 2A). However, a notably higher DNA concentration was observed in the maintained [g−] condition compared to any of the plates transferred to the high-AQI site. This elevated DNA concentration was associated with an increased bacterial-to-fungal relative abundance in the maintained [g−] condition, indicating promoted bacterial growth and/or reduced fungal growth without gas exposure at the low-AQI site (Figure 2A).

To explore variations among the cultivable microbial communities resulting from differential exposure to site-specific pollutant gases, we examined their bacterial and fungal aerobiomes using high-throughput sequencing technology. We obtained a total of 1,628,799 and 1,652,292 high-quality sequences for bacterial 16S rRNA and fungal ITS genes, respectively, resulting in 396 bacterial and 324 fungal ASVs. To ensure representative ASVs from each condition, we selected ASVs identified in at least two out of three biological replicates for subsequent analyses. This yielded 113 bacterial and 43 fungal identified ASVs. Additionally, to isolate the impact of the pollutant gases between sites on the cultivable communities and exclude the influence of other environmental differential factors between sites like temperature, the abundance of ASVs in each gas-exposed community (g+) was standardized based on their counterparts’ non-gas-exposed communities (g−) for all posterior analyses (Tables S1 and S2). Our results revealed a significant increase in the alpha diversity (the number of observed features and the Shannon index) of the bacterial and fungal communities exposed to gases (relative to non-exposed) when transferred to the high-AQI site compared to those maintained at the low-AQI site (Figure 2B). Furthermore, in the beta diversity results, the principal coordinates analysis (PCA) revealed that bacterial and fungal replicates within each condition clustered together in space and separated from the replicates of the other condition (Figure 2C). These results indicated that, despite the common origin of the microbial communities (collected from the low-AQI site), the differences in the concentration of some gases between the low- and high-AQI sites (and not in the meteorological conditions) significantly affected the diversity of the cultivable bacterial and fungal aerobiomes.

### 3.3. Taxonomy and Imputed Functions of the Aerobiome-Cultivable Communities Are Modulated by Environmental Gaseous Pollutants

**Figure 3:**
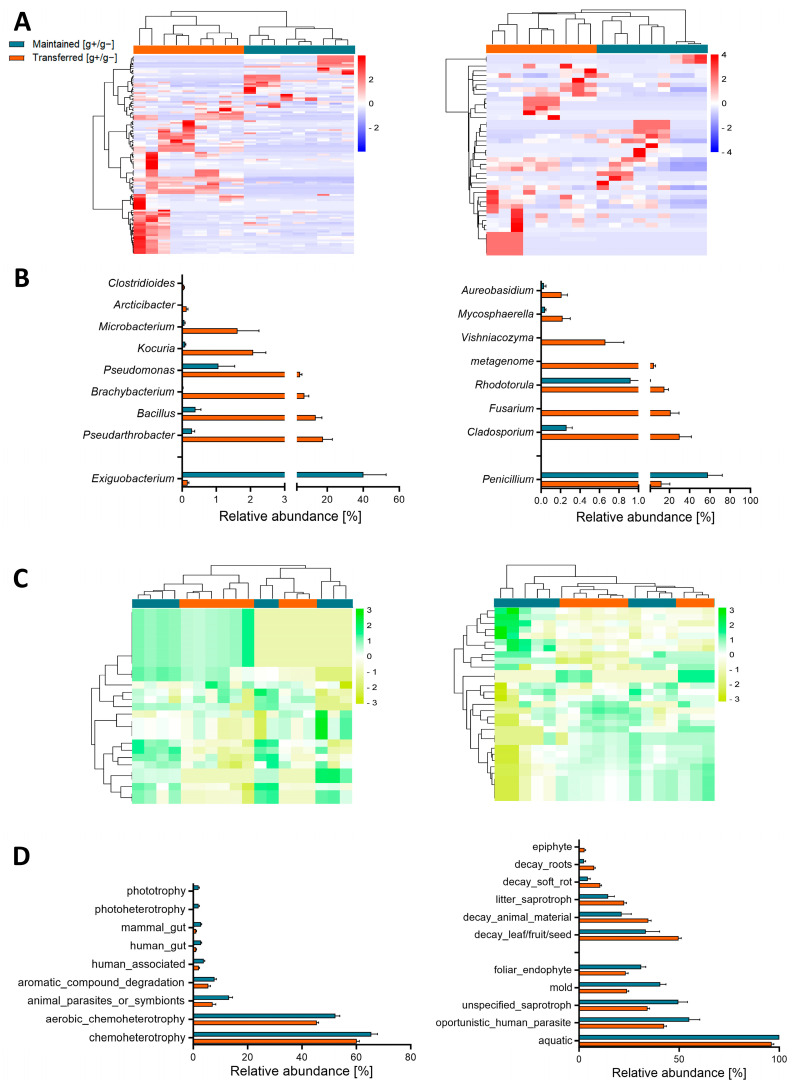
Taxonomic and imputed functional analysis. (**A**) Bacterial (**left**) and fungal (**right**) Z-score hierarchical cluster based on the Euclidean distance of the ASV composition using the g+/g− ratio data. (**B**) Bacterial (**left**) and fungal (**right**) genera significantly different between the maintained and transferred conditions using the g+/g− ratio data (Mann–Whitney; *p* < 0.05). (**C**) Bacterial (**left**) and fungal (**right**) Z-score hierarchical cluster based on the Euclidean distance of the imputed functional composition using the g+/g− ratio data. (**D**) Bacterial (**left**) and fungal (**right**) imputed functions significantly different between the maintained and transferred conditions using the g+/g− ratio data (Mann–Whitney; *p* < 0.05). Maintained [g+/g−] (blue): samples collected from the low-AQI site and grown at this same site; transferred [g+/g−] (orange): samples collected at the low-AQI site and grown at the high-AQI site. g−: No gas exposure; g+: gas exposure. Z-score hierarchical clusters were performed on the online https://www.bioinformatics.com.cn/ (accessed on 1 February 2023) platform.

The cultivable bacterial and fungal communities maintained at the low-AQI site or transferred to the high-AQI site responded differentially to variations in pollutant gases, showing taxonomic distinctions at all levels. Similar to the beta diversity findings, a hierarchical cluster analysis of the overall bacterial and fungal cultivable communities at ASV abundance level revealed a perfect separation between samples exposed to different pollutant gases (Figure 3A), highlighting the impact of gases on the specific taxa of airborne bacteria and fungi. At the phylum level, four bacterial and three fungal phyla were identified (Figure S3A,C), revealing a significant over-representation of Actinobacteriota and Basidiomycota in the samples transferred to the high-AQI site, while Proteobacteria were more abundant in the samples maintained at the low-AQI site (Figure S3B,D). At the genus level, 39 bacterial and 13 fungal genera were identified (Tables S1 and S2). The significantly over-represented taxa were predominant in the community transferred to the high-AQI site (*Pseudarthrobacter*, *Bacillus*, *Brachybacterium*, *Pseudomonas*, *Kocuria*, *Microbacterium*, *Arcticibacter*, and *Clostridioides* bacterial genera; *Cladosporium*, *Fusarium*, *Rhodotorula*, *Vishniacozyma*, *Mycosphaerella*, and *Aureobasidium* fungal genera) (Figure 3B). Remarkably, only the bacterial genus *Exiguobacterium* and the fungal genus *Penicillium* exhibited significantly higher abundances in the samples maintained at the low-AQI site, with both genera having the highest overall relative abundances in the communities (Figure 3B). These findings suggest that these genera are sensitive to variations in the concentrations of NO and/or O_3_ in the air.

Finally, to explore the relationship between variations of polluting gases and predicted functional capacities, we examined the imputed biological functional profiles within the bacterial and fungal communities across the samples. Opposite to the clusters formed from the relative abundance of the taxonomic composition, the hierarchical cluster analysis of the biological functions did not cluster all the samples from the same condition (Figure 3C). However, we observed significant differences in imputed functions within both bacterial and fungal communities between conditions (Figure 3D). Notably, all of these identified bacterial imputed functions were significantly over-represented in the samples maintained at the low-AQI site (including chemoheterotrophy, aerobic chemoheterotrophy, animal parasites or symbionts, aromatic compound degradation, human-associated, human gut, mammal gut, photoheterotrophy, and phototrophy). In contrast, the fungal community exhibited the over-representation of imputed functions in both gaseous differential conditions, with aquatic, opportunistic human parasite, unspecified saprotroph, mold, and foliar endophyte over-represented in the communities maintained at the low-AQI site, and decay leaf/fruit/seed, decay animal material, litter saprotroph, decay soft rot, decay roots, and epiphyte over-represented in the communities transferred to the high-AQI site, highlighting the impact of air gases on both the taxonomy and functions of the microbial communities of bacteria and fungi in the cultivable aerobiome.

## 4. Discussion

Research on the aerobiome and its association with air pollution has mainly focused on the influence of particulate matter (PM) on the bacterial community [10,30], with a limited focus on the fungal community and overlooking the effects of gaseous pollutants. In this study, we examined the effects of different ambient gaseous pollutants on the total bacterial and fungal cultivable communities from a common source using HTS. We evaluated the cultivable communities, rather than the entire aerobiome community, because cultivation offers the possibility of experimentally studying the adaptation of microorganisms to different stimuli, such as variations in gaseous pollutants. The environments selected for the study were two contrasting districts in the historical AQI of the Metropolitan Region of Chile (RM), Las Condes (low-AQI site) and Pudahuel (high-AQI site) (Figure 1A). As expected, during our winter experimentation week, the high-AQI site showed significantly lower concentrations of ozone (O_3_) and a higher abundance of nitric oxide (NO) compared to the low-AQI site (Figure 1C), as historically has been observed in these districts (Figure S1B). Interestingly, nitrogen oxides, produced via the combustion of fossil fuels in power plants and vehicles [31,32], are involved in O_3_ formation but can paradoxically also degrade O_3_ [33]. Thus, areas with lower fossil fuel combustion may lead to increased O_3_ concentrations due to less O_3_ degradation in less-polluted environments, an observation that aligns with the observation at our low-AQI site.

Since we aimed to isolate the impact of pollutant gases on the cultivable communities, we standardized the effect of gases with non-gas-exposed communities, similar to what has been applied in other microbial community studies [24,25]. This approach ensured an unbiased and more specific analysis of our data, considering the observed variation in meteorological factors among sites, like temperature (Figure 1D), a known driver of the environmental microbial structure [34,35]. Therefore, using the experimental and analytical approach developed, we could associate changes in the cultivable microbial community of the aerobiome specifically with variations in O_3_ and NO concentrations. While it is widely known that O_3_ has antimicrobial properties [36,37,38,39] and that NO is highly toxic to microbial life due to its rapid reaction with free radicals and transition metals [40,41,42], limited studies have been reported on the effect of these gases on the bacterial and fungal community of the aerobiome [9,10,13,17,30]. Interestingly, our results indicate that the samples exposed to gases tended to decrease the concentration of total DNA (suggesting a lower number of total microorganisms), which is particularly evident in the communities maintained at the low-AQI site without exposure to gases compared to the communities transferred to high-AQI site exposed to its gases (Figure 2A). Moreover, the exposure to gases showed a lower richness (observed features) in the bacterial and fungal communities (medians of less than 1), particularly in the samples that were maintained at the low-AQI site (Figure 2B). On the other hand, the diversity increased for both cultivable bacterial and fungal communities when transferred to the high-AQI site compared to those maintained at the low-AQI site, indicating that increased NO and/or decreased O_3_ promoted the growth of microorganisms unable to grow at the low-AQI site. This increase in diversity can be explained primarily by the growth of O_3_-sensitive microorganisms but also by the finding that some microbial taxa are capable of harnessing energy through the reduction of NO [43].

At the taxonomic level, the observed changes in the community structure of the aerobiome were clearly reflected in the segregation of hierarchical clusters between samples maintained at the low-AQI site or transferred to the high-AQI site (Figure 3A). Furthermore, our results showed that the genera driving the compositional changes of the cultivable aerobiome were predominantly over-represented in the samples transferred to the high-AQI site compared to the samples maintained at the low-AQI site (eight bacteria and six fungal genera) (Figure 3B). Many of these over-represented bacterial and fungal genera encompassed human pathogen species [44,45,46,47,48,49,50,51,52]; therefore, changes in their abundance due to pollution may significantly worsen human disease outbreaks. On the other hand, only the bacterial genus *Exiguobacterium* and the fungal genus *Penicillium* displayed significantly higher abundances in the maintained than transferred communities, and they both also had the highest relative abundances (Figure 3B). Remarkably, species from both genera have shown a higher resistance to O_3_ compared to other species [53,54,55], while currently, only *Penicillium* has members with increased sensitivity to NO [56]. Interestingly, other authors have also reported correlations between the aerobiome composition (richness and/or abundance) and the environmental O_3_ and NO concentration. For example, Li and co-workers [9] reported a negative correlation between the O_3_ concentration and the abundance of *Bacteroides*, *Subdoligranulum*, and *Clostridium_sensu_stricto_1*, as well as a positive correlation with *Solirubrobacter* and RB41 in the urban aerobiome of central China. Additionally, Núñez and García [14] informed a significant negative correlation between Firmicutes’ abundance and the NO concentration in the urban environment of Madrid (Spain). Although these studies were conducted without experimental interventions characterizing the aerobiome independent of culture, they show interesting correlations between the concentration of these environmental gases (and other meteorological factors) and the abundance of some microbial taxa in the aerobiome.

Finally, at the functional level, the hierarchical cluster analysis indicated that the microbial imputed functions did not separate the different gaseous environmental treatments (Figure 3C), suggesting that despite the microbial taxonomic structure’s sensitivity to environmental changes, there appears to be ecosystem multi-functional resilience within the microbial community [57]. However, significant differences in imputed functions within both bacterial and fungal communities among conditions (maintained or transferred) were observed (Figure 3D). Remarkably, the nine differential bacterial imputed functions were all over-represented in the communities maintained in the low-AQI site. Therefore, despite only one bacterial genus being over-represented at the low-AQI site (*Exiguobacterium*, Figure 3B), its high abundance seems to generate a crucial impact on the distinctive biological functions of the community. In fact, this genus has members implicated in all the imputed functions over-represented at the low-AQI site, such as phototrophy [58], chemoheterotrophy [59], aromatic compound degradation [60], animal parasites or symbionts [61,62], and human-associated [63]. Regarding the fungal cultivable community, imputed functions were over-represented in both gaseous differential conditions (six at the high-AQI site and five at the low-AQI site; Figure 3D). When exposed to the differently concentrated gases present at the high-AQI site, fungi appear to distribute the community functions among various members, many of which are linked to the decomposition of organic substances. Thus, air pollution may be an important factor affecting microbial community composition by impacting environmental nutrient cycle recycling through the process of decomposition. On the other hand, at the low-AQI site, only the *Penicillium* genus appears to play a key role in community functionality due to its over-representation (Figure 3B). This highly abundant genus in the communities maintained at the low-AQI site includes species known for various over-represented functions at the same site, such as being aquatic [64], opportunistic human parasites [65], saprotrophs [66], and foliar endophytes [67]. 

This study represents the first comprehensive assessment of whole cultivable communities of bacteria and fungi via amplicon HTS in an experiment in which changes in the composition of the same original microbial community were assessed as a function of differences in two pollutant gases at the environmental level. While acknowledging the potential influence of unmeasured gases, the study establishes certainty that at least part of the changes observed in the community can be attributed to O_3_ and NO, which provides the basis for future studies on the individual effect of each of these gases on specific microbial taxa and their impact on the aerobiome of urban environments.

## Data Availability

All sequence data used in this study have been deposited in the Sequence Read Archive (SRA) of the National Center for Biotechnology Information (NCBI) under the BioProject accession number PRJNA1064435.

## Supplementary Materials

The following supporting information can be downloaded at https://www.mdpi.com/article/10.3390/microorganisms12061103/s1: Figure S1: Historical AQI and individual contaminants’ values; Figure S2: Representative microbial communities from the study; Figure S3: Bacterial and fungal phylum taxonomic analysis; Table S1: Bacterial ASVs’ taxonomy and relative abundance using the g+/g− ratio data; and Table S2: Fungal ASVs’ taxonomy and relative abundance using the g+/g− ratio data.
