# Supplementary material for: The Impact of Environmental Gaseous Pollutants on the Cultivable Bacterial and Fungal Communities of the Aerobiome
Source: Microorganisms. 2024 May 29;12(6):1103. doi: 10.3390/microorganisms12061103 (PMC11206153; doi:10.3390/microorganisms12061103)
Supplement: Supplementary file 1 [file microorganisms-12-01103-s001.zip › Supplementary Material_Mej¿¬as et al 2024.pdf]

Supplementary Material

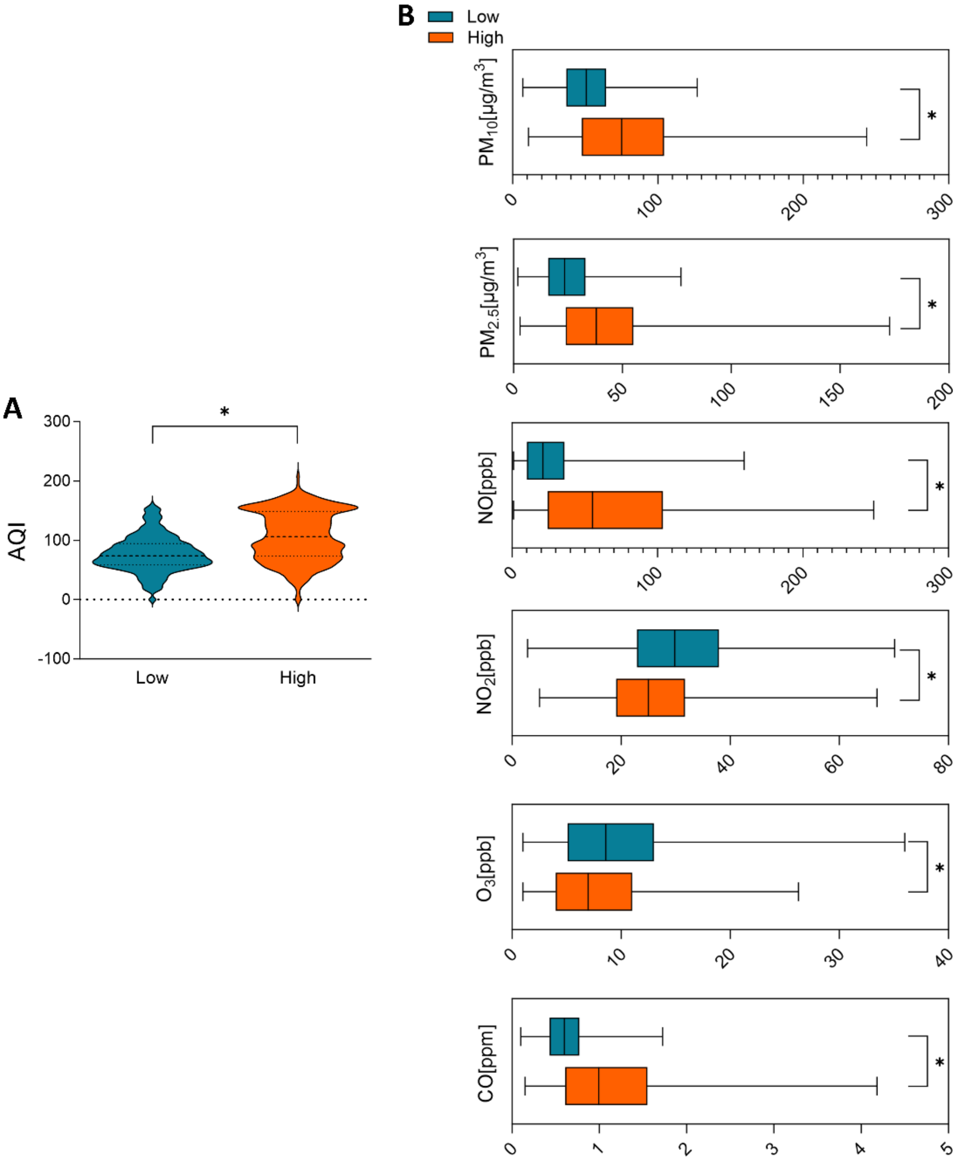

Figure S1. Historical AQI and individual contaminants' values. A. Violin plots of the historical Air Quality Index (AQI) data (from 2013 to 2022) obtained from Sistema de Información Nacional de Calidad del Aire (SINCA; https://sinca.mma.gob.cl/) from the low and high AQI sites. Dashed bold lines represent the median. Dotted lines at the tops and bottoms represent 75th and 25th quartiles, respectively. Asterisk indicates statistically significant differences between sites (Mann-Whitney; $p < 0.05$). B. Box plots of the historical individual contaminants' values (from 2013 to 2022) obtained from SINCA. Horizontal lines within boxes represent the median. The tops and bottoms of the boxes represent 75th and 25th quartiles, respectively. Asterisks indicate statistically significant differences between sites (Mann-Whitney; $p < 0.05$).

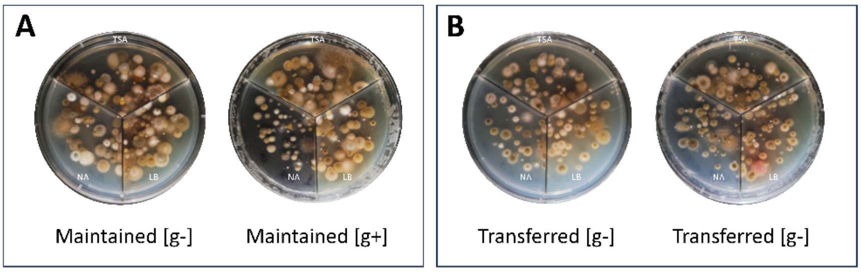

Figure S2. Representative microbial communities from the study. A. Cultivable communities collected from Las Condes (low AQI site) and grown in this same site **(maintained)** during the experimentation week (August 12th to 19th, 2022). B. Samples collected from the low AQI site and grown in the high AQI site **(transferred)** during the experimentation week. NA: Nutrient Agar, LB: Luria Bertani, TSA: Tryptic Soy Agar. g-: No gas exposure; g+: gas exposure.

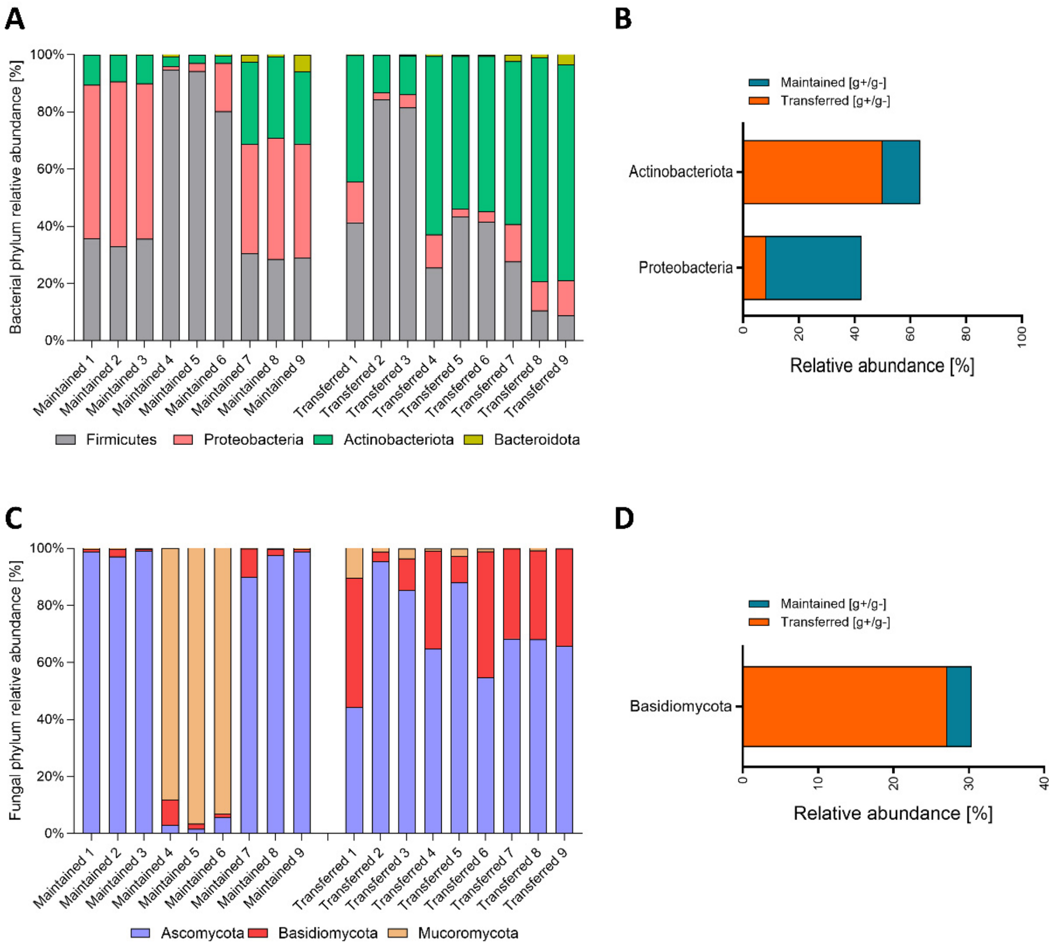

Figure S3. Bacterial and fungal phylum taxonomic analysis. A and C. Bacteria and fungal relative abundance at phylum taxonomic level, respectively, using the g+/g- ratio data. B and D. Bacterial and fungal phyla significantly different between the maintained and the transferred conditions using the g+/g- ratio data (Mann-Whitney; $p < 0.05$). Maintained [g+/g-] (blue): Samples collected from the low AQI site and grown in this same site; Transferred [g+/g-] (orange): Samples collected in the low AQI site and grown in the high AQI site. g-: No gas exposure; g+: gas exposure.
